# Orthorexia nervosa: Healthy habit or pathology? Experiences and expansion of the consciousness of the correct diet

**DOI:** 10.1016/j.heliyon.2025.e42254

**Published:** 2025-01-27

**Authors:** Ginés Mateo-Martínez, Antonio Vázquez-Sellán, María Luisa Díaz-Martínez, María Carmen Sellán-Soto

**Affiliations:** Nursing Department, Faculty of Medicine of the Autonomous University of Madrid, Spain

**Keywords:** Orthorexia nervosa, Nursing, Mental health, Eating disorders, Qualitative research

## Abstract

**Aim:**

Exploring the lived experience of women concerned about following a healthy and sustainable diet: subjectivity, ideas, descriptive concepts, and constitutive strategies.

**Method:**

Based on the epistemological paradigm of phenomenology, the theoretical perspective of symbolic interactionism, and the methodological perspective of phenomenological reduction, a qualitative discourse analysis was performed through different strategies (thematic content analysis and interpretive phenomenological analysis, applying *ad hoc* techniques).

**Findings:**

The phenomenological field of healthy eating intermittently unfolds in both experience and discourse, akin to a process of expansion and contraction, in the narrative “Expanding the Consciousness of Healthy Eating”. This encompasses two main themes: (1) *Researching and Gathering Information*, with the subthemes ‘Health Socialisation,’ ‘My Purchase: Assessing Food Reliability,’ ‘Moral Burden,’ and ‘Compensation as a Mechanism of Channelling Deviation’; and (2) *Becoming Healthier*, with the subthemes ‘Highlighting Benefits,’ ‘Marketing as a Pandemic Resource of Ideals,’ and ‘Is the Clinical Eye of the Family a Stigmatising Label?'.

**Conclusion:**

The process of becoming healthier entails a struggle with the environment and with one's inner self. This struggle is open to the entire world and guided by the unalienable tenets of health, respectfulness, goodness, and beauty as ‘the only alternative’ to the suffering caused by a conventional diet.

## 1 Introduction

Within the realm of the commodification of images, and aesthetic and ethical social ideals (such as utmost beauty, extreme health, “*top cancer-fighting foods*”, “*the best foods for weight loss*”, “*rejuvenating foods*”, “*life-changing product*”, and multiple emphases on healthy lifestyles), an omnipresent question emerges and is prominent for its scope and coverage through technological information and communication devices: the modern tyranny of health [1,2]. Specifically, the sacred value of health for any human being is now diminished by a crisis of values in the context of globalisation, simultaneously generating a clear *butterfly effect*, as shown by a marked fragmentation at all levels. In other words, the right to health comes into conflict with the commodification of health to the highest bidder (health commodified by the pharmaceutical industry, among other large capital companies, to the detriment of a holistic conception of well-being), resulting in dehumanised health services and misinformation [[3], [4], [5]]. Thus, the proliferation of “healers” or “health vendors”, who delve into self-management and self-learning, contributes to confusion and the unhealthy worship of health. Furthermore, the commodification of health is particularly relevant and complex when observed through the lens of education: education and health are linked because the former mediates the perception of the latter [6,7].

Excessive interest in, concern about, or obsession with healthy food was first defined by the physician Steven Bratman, who coined the term “orthorexia nervosa” in 1997. The word orthorexia etymologically derives from *ortos* (correct) and *orexis* (appetite), that is, healthy eating. Bratman argues that orthorexia nervosa is an obsessive eating disorder considered healthy by those with this condition, which sometimes leads to a state of malnutrition and even death [8]. This definition represents the initial conceptualization and theoretical foundation upon which orthorexic behaviours are recognized as a health problem.

Several factors may promote the instability of the orthorexia nervosa construct, which is linked, through differential diagnosis, with well-known and taxonomically defined disorders, that is, with their own descriptors and defining criteria, such as chronic delusional disorder, anorexia nervosa, and obsessive-compulsive disorder. Thus, chronic delusional disorder is ruled out because the philosophical and religious ideas and values of natural life in orthorexia nervosa, although often irreducible to logical argumentation, are not established through delusional thinking. Furthermore, anorexia nervosa is ruled out, as weight recovery does not improve the obsessive symptoms, and the patient exhibits neither anxiety nor fear of progressive weight gain, body image distortion, an unlimited desire for thinness, or purgative behaviours, at least not habitually. However, a history or episode of an eating disorder (especially anorexia nervosa) is a strong determinant of the onset of orthorexia nervosa, suggesting that this emerging disposition may be found within the spectrum of traditional eating disorders [9,10].

In any event, some studies emphasise the symptomatic relationship between orthorexia nervosa and obsessive-compulsive disorder [11]. According to Bratman and Knight [8], these two conditions are linked because obsessive-compulsive manifestations partly overlap with some orthorexic patterns, such as somatisation disorder and hypochondriasis (persistent, obsessive, health-related intrusions, fear of developing a life-threatening disease, panic over strange somatic symptoms, which are associated with a serious, life-ending illness, and checking various bodily functions, such as heart rate, breathing rate, body temperature, or various aspects of body or anatomical image); perfectionism (self-demand, concern about minor and irrelevant details, an urgent need to perform tasks perfectly, an extreme need to know or remember trifling matters, and maintaining things in perfect order, among other patterns); the philosophical disposition (somewhat uncertain tendencies, experience of an inextricable metaphysical process); and, perhaps most demonstratively, the characteristic preoccupation with health and physical appearance through deviations related to diet, physical exercise, lifestyle, fashion, personal image and anti-aging [[12], [13], [14], [15], [16]].

Orthorexia nervosa is not formally recognized by any classification manual or renowned source, at least not in an explicitly and exhaustively structured manner based on differential diagnostic criteria. Neither the International Classification of Diseases, Eleventh Revision (ICD-11), nor the Diagnostic and Statistical Manual of Mental Disorders, Fifth Edition (DSM-5), address orthorexia nervosa separately from other disorders and diseases; however, its inclusion in DSM-5, in the section of eating disorders, has been discussed but ultimately not specified. Thus, professionals who use this diagnostic manual consider orthorexia nervosa an unspecified eating disorder. Therefore, classification manuals sometimes serve as “catch-alls” to temporarily place maladjustment symptoms involving the affected individual and their environment simultaneously [17,18].

Some studies have indicated the prevalence of orthorexia nervosa in the general population, albeit calculating this value in small samples and with highly defined profiles. The prevalence of orthorexia nervosa is approximately 6.9 % in the general population and ranges from 35 to 57.8 % in high-risk groups (healthcare professionals, athletes, and artists) [19,20]. However, in Spain, some specialists report that 0.5–1% of individuals who visit their practice present with orthorexia nervosa [21].

Comparing epidemiological results requires conducting studies with more representative samples and more accurate measuring instruments [22,23]. Thus far, several studies have compromised the reliability and validity of the ORTO-15 questionnaire. Initially developed by Bratman himself, this self-report diagnostic measure has undergone modifications in various attempts at its validation in other languages and in its psychometric development aimed at identifying psychosocial factors and comorbid psychopathology [24]. Consequently, due to insufficient research (ambiguity and variability in the results) and to the multifactorial nature of orthorexia nervosa, this condition must be addressed using a qualitative study approach [25]. In fact, some studies advocate for an empirical-holistic approach and an interpretative analysis [13,26]. Specifically, the objective is to explore the experience of individuals with orthorexia nervosa (concern about or obsession with healthy eating).

## 2 Materials and methods

### 2.1 Design

To approach the lived experience, that is, to understand the subjectivity related to the phenomenon under study (concern about following a healthy and sustainable diet), qualitative research focuses on the words of individuals, spoken and/or written, and on observable behaviour to generate descriptive data. Similarly, a phenomenological perspective is crucial for qualitative methodology because researching, ultimately, consists of analysing things from different points of view and delving into the subjective world of human experience for its understanding [27].

The search for and construction of knowledge from a phenomenological perspective are in line with the proposed objective of this descriptive study to approach lived experience. Furthermore, the crux of a descriptive phenomenological study lies in the relevance of the participants’ subjective experiences and in formulating research objectives in descriptive terms. However, the classification of phenomenological studies includes interpretive phenomenological studies (just as participants interpret and shape their lived experiences, the researcher must also interpret results through a hermeneutic lens in the knowledge construction process). Accordingly, the present study adopts a mixed (descriptive and interpretive) phenomenological approach to describe subjective experience. This approach stems from participant-researcher interaction and dynamics in methodological decision-making for understanding the phenomenon [28].

The theoretical-methodological perspective serves as both reference and foundational pillar whereby the researcher elucidates the phenomenon under study and conducts the research process. In the present study, the theoretical framework that sheds light on and is coherently aligned with the objective and epistemic paradigm of phenomenology is phenomenological sociology, more specifically, the sociological proposition of symbolic interactionism. This framework enables the researcher to methodologically apply phenomenological reduction as a tool to grasp the essence of subjective experience, emerging through an ever-changing and dynamic intersubjectivity [27,29].

### 2.2 Selection of informants

The research was conducted in the Autonomous Community of Madrid. Initially, any space or organisation promoting healthy eating (blogs and online forums) or selling healthy food (organic and sustainable food retailers, establishments and associations, herbalists and homeopathy centres) could have been valid. After an ambitious and repeated search for potential spaces that could contribute data to the study and several unsuccessful attempts at establishing contact, communication was eventually made with one of them: M. F. CÁTERING ECOLÓGICO, S.L. At the registered office of this limited company, the researcher was allowed to access and gradually become involved and participate in the phenomenon under study.

The inclusion criteria for selecting study respondents and social actors were defined based on the richness and contribution of meanings of the data, focusing on experiential aspects to comprehensively examine the phenomenon.

Initially, the following inclusion criteria were established for the study population:

a. Men or women diagnosed with orthorexia nervosa.
b. Men or women obsessed with healthy eating.

These inclusion criteria were reformulated and ultimately defined as follows:

a. Women concerned about following a healthy and sustainable diet.
b. Women who, without having necessarily been diagnosed with orthorexia nervosa, maintain a lifestyle based on healthy food.

Modifications to inclusion criteria are justified according to the evolution of the research process and the emergence of data redirecting the analysis and methodological decisions. From the outset (the initial literature review), limited access to individuals with a biomedical diagnosis of orthorexia nervosa was anticipated given the emerging and relatively novel nature of this phenomenon, its online presence in the media, and its instability as an ambiguous social construct, among other reasons. This human condition is assessed from two different perspectives: some pundits argue that the predisposition to healthy eating fits within the realm of eating disorders (although orthorexia nervosa does not appear as such in diagnostic manuals, from this perspective, this condition should be included in unspecified eating disorders, according to DSM-5). Conversely, others merely consider orthorexia nervosa a lifestyle.

In this research, purposive sampling was used to access the study area (deliberate selection of informants based on accessibility, that is, easy location and access) [30]. However, access to similar study areas (other spaces and organisations advocating a healthy and sustainable diet) was limited, thus requiring an alternative selection strategy. Accordingly, the participants were recruited by snowball selection (opportunistic access to new informants through other contacts) [31].

The methodological decision of theoretical saturation was influenced by the conviction and level of analytical induction achieved in this study. This influence enabled a comprehensive approach to the phenomenon; hence, its communication in descriptive terms derived from analytical inferences and inquiries, with consistent results. In this study, three participant observation sessions lasting between six and 7 h each, and four in-depth interviews were conducted with women concerned about following a healthy and sustainable diet^1^ (Table 1).

**Table 1:** Defining characteristics of the informants. Autonomous Community of Madrid.

Informants	Age (years)	Academic Level	Labor Performance	Conditioning
				Disease of Food
ML (most relevant social actor in participant observation)	50/55	University Studies	Administration and Nutrition	Cancer (in the past)
E	42	Primary Studies	Monitors Sports Center	Son with chronic metabolic disease
M (elite informant)	42	University Studies	Agricultural Engineering	Cancer (in the past)
MA	40/45	University Studies	Medicine	Not referred

### 2.3 Data collection

The researcher maintained an overt stance in participant observation, so all participants in this context were aware of and acknowledged the researcher's presence. Throughout the participant observation sessions, the data were primarily condensed into two axes to formulate the research objectives around ideas, concepts, and construction strategies of lived experience.

In line with previous studies on lived experience, this phenomenon was contextualised through qualitative interviews. The initial open-ended approach was subsequently remodelled and ultimately converted into a phenomenological in-depth interview format (Table 2). The decision to conduct in-depth interviews in later phases of the research process was influenced by several factors: the research interests became clearer and relatively well defined, and the researcher aimed at delving into the subjective human experience, beyond the present moment, by exploring narratives of past events and future expectations to understand the ideas and descriptive concepts of the experience and the construction strategies of lived experience [22,32].

**Table 2:** Guiding script for interviews.

Objectives	Bibliographic Concepts/Sensitive Concepts	Questions
To explore the experience of individuals with orthorexia nervosa (concern about or obsession with healthy eating)	Identity (the conception one has of oneself, shaped by personal characteristics, beliefs, values, and experiences that make an individual unique. It involves both individual factors (such as personality and abilities) and social factors (such as roles, culture, and relationships).	- What is it like to live with a lifestyle linked to healthy eating? - What things make it possible to lead a life of this type? - How did you get there and why did you decide to follow this path?
	Self image (the mental picture or perception one has oneself, including physical appearance, personality traits, abilities, and social roles. It reflects how individuals see and evaluate themselves).	- How does eating healthy make you feel? - What difference is there or did you perceive when you made the decision to start? - What are the aspects that best describe a person who eats exclusively healthy food?
	Worldview (the overall perspective through wich an individual or group interprets and understands the world, including beliefs, values, and assumptions about life, reality, and existence).	- How is your daily diet? Where? In what spaces or environments? - What do you think of the world in terms of food? - How do you understand that this issue is addressed by society or politics?
	Inside world (refers to a person's internal mental and emotional landscape, including their thoughts, feelings, desires, and perceptions. It encompasses the subjective experiences and inner life that shape how and individual understands and interacts with the world around them).	- How does eating healthy make you feel? - How would you describe your inner experience, your sensations and feelings?
	Society and food (the relationship between food and social structures, including how food practices, culture, production, and consumption are shaped by and influence societal norms, traditions, economics, and health. It explores the role food plays in social identity, community, and daily life).	- How are your social relations with respect to the diet you follow? What do they think? What do they tell you? - How do people who support your way of eating? - How does someone who decides to follow this diet learn? With which groups? Why?
To identify concepts and ideas that describe the experience	Feelings of well-being (refer to a sense of overall health, happiness, and contentment, characterized by positive emotions, life satisfaction, and a balance of physical, mental, and emotional health).	- Why do you think these foods are better than others? What do these foods have that others don't? - Why do you feel better eating these foods? How is that feeling of well-being?
	Healthy food (refers to foods that provide essential nutrients, such as vitamins, minerals, proteins, and healthy fats, while supporting overall physical well-being and reducing de risk of chronic diseases. These foods ar typically whole, minimally processed, and balanced in terms of nutritional content).	- What do you understand by healthy eating?
	Self control (the ability to regulate one's emotions, thoughts, and behaviors, especially in challenging situations, to achieve long-term goals or adhere to personal standards and values. It involves resisting impulses and maintaining discipline).	- How does healthy eating affect self-control, or vice versa? - How do you feel that you control yourself? In what aspects of your body or your being do you feel self-control?
	Disease prevention (refers to actions or measures taken to reduce the risk of developing diseases, aiming to maintaining health and avoid illness. It includes practices like vaccinations, healthy eating, regular exercise, and medical screenings).	- How is healthy eating related to the prevention of health problems? What theory do you rely on to think like this?
	Spirituality (refers to the pursuit of a sense of meaning, purpose, and connection to something greater than oneself, often involving beliefs, practices, or experiences related to the sacred, the divine, or the essence of life. It may or may not be tied to religious traditions).	- How does this lifestyle make you feel inside? Where does it take you? What do you hope to achieve deep down?
	Purification (the process of cleansing or removing impurities, whether physical, spiritual, o symbolic, to achieve a state of purity or clarity. I can involve rituals, practices, or treatments aimed at renewing or restoring purity).	- How is the consumption of healthy food related to cleansing or detoxing the body? Why this purpose of having a clean body?
	Ideology (a set of beliefs, values, and ideas that shape an individual's or group's worldview, guiding their social, political, or economic perspectives and actions. It provides a framework for interpreting and responding to the world).	- What ideals do you rely on to follow your diet? - How do dietary fads influence? - How is the ecological culture and this type of food related? - What is it like to follow a healthy diet as a woman/man? Why?
	Blame (the act of holding someone or something responsible for a fault, wrongdoing, or undesirable outcome. It involves assigning accountability or fault for a negative event or situation).	- What does it mean not to follow the planned healthy diet? - How do you manage if you do not strictly adhere to the diet?
To know strategies of constitution of experience	Worry/Obsession (the feeling of anxiety of concern about potential problems of future events, often accompanied by overthinking. Obsession is an intense, persistent preoccupation with a particular thought, idea, or activity, often to the point where it interferes with daily functioning).	- What makes you worry about food? - How does your concern relate to your character or way of being? - What does your family think? And your friends?
	Emphasis on food (refers to the importance or focus placed on food in various contexts, such as health, culture, of lifestyle, highlighting its role in nutrition, social practices, or personal identity).	- Why is the quality of food so important? - Why is diet the most important thing? What other things in your life are important? Are they related to your diet?
	The purchase (refers to the act of buying or acquiring goods or services in exchange for money or other forms of payment).	- How do you buy food? In what places? What is the most important thing when buying? What are you looking at? Why?
	Food preparation (the process of getting food ready for consumption, which includes tasks like cleaning, cutting, cooking, and seasoning ingredients to create a meal).	- How do you cook the food you buy? - What means and utensils do you use to cook? Why?
	Food consumption (refers to the act of eating or drinking food and beverages to nourish the body and satisfy hunger or thirst).	- What is lunch time like? How is the act of food intake? Who are you eating with? What do you think while you eat? - What is the relationship between shopping, cooking and eating healthy food?
	Food restriction (refers to deliberate limitation or reduction of food intake, either in terms of quantity, specific types of food, or calories, often for health, medical, or personal reasons).	- Why are there foods that are restricted? What are those foods? For what purpose are they restricted? What do you get?

The in-depth interviews lasted between 60 and 120 min and were recorded, transcribed, and supplemented with simultaneous notes (clarifications, doubts, interpretations, and inferences) on verbal and nonverbal expressions and on the female informants’ speech process.

### 2.4 Data analysis

In line with the theoretical-methodological orientation (symbolic interactionism and phenomenological reduction, respectively) and in the context of non-standardised qualitative research methods, the analysis was conducted based on a framework categorising analysis techniques according to the flow and orientation of emerging data. This *ad hoc* approach to analysis is a way of eclectically generating meaning through words, numbers, figures, flowcharts, or their combination [32].

The theoretical framework adopted in this study was based on Phenomenological Sociology, which embraces the process of intersubjectivity as a source of knowledge and understanding of the phenomenon; furthermore, its analytical approach rests upon the set of phases of traditional phenomenology, within an intersubjective relational system of reflexivity and methodological, conceptual, comprehensive and theoretical information sharing between the researcher and the participants. In this sense, the data analysis involved several stages according to the methods used in this *ad hoc* approach to analysis. Nevertheless, this analysis was aligned with the dynamics proposed by Miles and Huberman [33], delineating the following phases of the analysis process: data reduction, data display (organisation and emergence of the emerging theory), and conclusion drawing and verification (Appendix A). These phases were guided by three overarching data manipulation domains: discovery, coding, and contextualisation [27,32].

Building upon the phenomenological field of healthy eating to theorise the phenomenon [34], Margaret Newman's foundational framework of *Health as expanding consciousness* was adopted in this study. In this context, the significance lies in recognising patterns in the process of expanding consciousness [35]. The theory was extensively used to explore and understand the health experience within the illness, based on the fundamental premise that crisis situations can catalyse and facilitate movement towards higher levels of consciousness [36].

### 2.5 Ethical aspects

The conduct of the research and production of the report met the criteria established in the *Consolidated criteria for reporting qualitative research* [37]. Research consent was obtained from the corresponding body: Research Ethics Committee of the Faculty of Medicine of the Autonomous University of Madrid (CEI-69-124).

Throughout the research process, both maximum and minimum ethical standards in research with human beings were considered [38,39]. Below, each principle is outlined along with the interventions undertaken to ensure their consideration in the process: autonomy (informed consent); justice (opportunity for participation based on theoretical sampling); and beneficence/non-maleficence (creation of a comfortable and warm work environment conducive to data collection, relevant question formulation, and emotional sensitivity towards the participants and the ambiguous researcher's role). To ensure compliance with these ethical principles, efforts were made to justify the questions, practice active listening and empathy, clarify the researcher's role, maintain personal data confidentiality, and guarantee voluntary participation.

From the outset of the research process, the informants struggled to open up to and establish a rapport with the researcher if they were informed that the study involved exploring the concept of orthorexia nervosa. Therefore, they were initially informed about only a vague interest in the phenomenon of concern about healthy eating so that later, with a stronger connection and openness, the diagnostic proposal could be addressed without violating the ethical principle of beneficence/non-maleficence.

The informed consent form was completed and signed by the four women who participated in the in-depth interviews. Additionally, in the context of participant observation, the gatekeeper informant also signed an informed consent form for the researcher's immersion in the field.

To uphold the principle of justice, a methodological decision was made to consistently pursue theoretical saturation, ensuring equal opportunity for all women to express their experiences. This approach aimed at facilitating understanding and knowledge of the phenomenon, thereby enabling its comprehensive representation and portrayal in the results.

### 2.6 Rigour

All quality criteria in qualitative research proposed by Guba [40] and by Castillo and Vásquez [41], namely credibility, transferability, reliability, and reproducibility, were met by working from different angles.

Thus, to ensure the quality of the work, a series of strategies were implemented to uphold scientific rigor in qualitative research. The principles of intersubjectivity, flexibility, and dynamism were embraced in the research process to assess its quality both throughout the process and in terms of outcomes. Throughout the process, continuous efforts were made to enhance conceptual clarity and methodological coherence. In terms of outcomes, the researcher's interpretative exercise and abstraction levels were acknowledged whilst striving for originality, credibility, relevance, applicability, and reproducibility.

Methodological rigor was upheld by the foundational concepts of the study. In other words, the focus was maintained on the research question while searching reflexively, interpreting, and making methodological decisions from the emergence of the first hypotheses, through those developed during the process, to the redefinition of the initial objectives, ultimately ensuring methodological and terminological consistency. Thus, the inherent dynamism of qualitative research navigates between field requirements and the researcher's limitations and capabilities. Within the intersubjectivity of the field and the inherent research process, the researcher's reflexivity renders the researcher a research instrument. Furthermore, from the researcher's subjectivity and methodological choices, observational, analytical, and comprehensive work with the data gives rise to theoretical directions suggested by the field of study.

To ensure the credibility of the results, the researcher communicated with them to verify the coherence of their representation with the subjective experience of the female participants by sharing and discussing the findings with some of the participants to assess their accuracy.

## 3 Findings

### 3.1 Expanding the consciousness of healthy eating

Based on the theoretical proposal of *Health as expanding consciousness*, themes related to the expansion of the concern about following a healthy and sustainable diet are presented in this study. These themes run parallel to the information and research process. By interpreting the meaning and expansive evolution of orthorexia nervosa, the condition is described as a constant exercise of survival and expansion.

As transverse axes to the phenomenological field of healthy eating, two predispositions synergistically evolve throughout the exposition of the condition by the female informants: on one hand, being informed and strongly predisposed to research and update knowledge, strategies, and resources; and on the other hand, a trend sometimes regarded as an expansion of the healthy condition, a phenomenon with repercussions in various contexts within health socialisation, primarily among family and friends.

*I think that today there are more people concerned about the issue than a few years ago. I don't know if because of all the diseases we have now … This is what worries people. That's my opinion***(MA_40/45)**. *I think it's evolution as a person, okay? It is your personal growth. There are times when I look at myself and I don't recognize myself. That is, I do not recognize myself as I was before. Probably … But that happens to all of us. […] Well, yes, it has changed. Now I was worried about things that didn't worry me before. And then you start to worry about food at the health level; You also have information because of the training you have. [ …] Of course, the way of constructing thoughts is different. […] And I perceive it as something positive. I suppose this will happen to everyone, although it depends on the phase they are in, of course … […] First you get the guilt syndrome, then rejection. Wow, this can't be; you go through a series of phases: you go up, you go down, you come back and then you realize …***(M_42)**.

#### 3.1.1 Researching and gathering information

Across the phenomenological field of healthy eating, that is, from the past subjective experience, through the present experience, to the envisioned future experience; the female informants show a predisposition to be informed about everything related to the condition (concepts, ideas, organisations, and training sessions, among others) and to research or exhaustively search for arguments supporting the plausibility and relevance of the ideological baggage and knowledge on healthy eating.

In this context, for the female informants, “having enough information” means having enough knowledge to assess and evaluate the reliability and quality of the food that is sold; being able to decide and choose adequately, in line with their beliefs and ideology; and feeling less deceived when making a purchase or encountering conflicting information.

*Ugh … Currently, you have to be very sure, very sure of what you are going to eat. Because not everything they sell you is quality. I come back to the same thing: they are cheating you. They are deceiving you, they are selling you a product. They tell you that it is the best quality it has, that it is the best on the market; and then you start to investigate that product a bit and it is not good; it's not good … it's all, it's all chemistry and so on. And in the end you are not eating healthy. They are poisoning you, that's for sure. There are many processed foods, which are sold to you as healthy and are not healthy. You have to be very careful there. Because look, bad luck, today there are few, very few natural foods***(E_42)**. *Carbohydrates are what make me feel happy … It's just sweet! It's the sweetness, I mean, it's all researched, you know? It is the sweetness, it is the affection; So you compare the food to all of this, and you do a little more research, because you already know how it's related, right? […] So, for me, eating like this is my daily life, my way of life. And I think everyone should do this … There's nothing that gives me pleasure in what I'm doing, you know? Say: I'm fine, I eat organically. No, I think it is my obligation. No, it is not an obligation, but it is the duty that we should all have, right? You don't have the information, you can't choose. The important thing is to have the information so you can choose***(MA_40/45)**. *Training is for people to make a change in their lives. You say: I want to eat organic, where do I start? Should I go to the supermarket to buy some organic spaghetti? Well, it's a start … […] The purists say: bah … But you, when you start to worry a little more, you say: and where does this come from? And the first thing people tell you: it's not very ecological if it comes from the other side of the world. So you say: uhm, you're starting to get interested. […] Before I could question the happiness of a person because of their diet, but now I say: do you have all the information?***(E_42)**.

#### 3.1.2 Health socialisation

The mode of health socialisation and healthy and sustainable diet is manifested in two more or less contradictory, yet simultaneous, aspects: on the one hand, the respondents demonstrated a willingness and desire to share subjective experiences in terms of expressing lived experiences, providing information and promoting ideas and messages endorsing the condition; on the other hand, they reported feeling deceived by the media and by the mechanisms of conventional commodification of food (including sustainable food). As a result, social gathering in defence of the condition is fostered, highlighting the challenges of strategic performance in daily life because the mechanisms responsible for the natural and ecological market process function inadequately.

*These meetings arise from the sports center where I go daily. It is in those places where this type of conversation and the subject of food are found the most***(E_42)**. *Of course, that makes it easier for you not to eat organic food outside the home. Or socialize, right? Because when you socialize … When I left home, I couldn't eat organic, because you can't find it, there isn't any in a restaurant. In some places I do it, hey, I have a glass of water and that's it. But in the end, since food also provides an energetic part, which is the good vibes you have with someone, it is the atmosphere. You change the meaning of food, right? If we get exoteric …***(M_42)**. *People will cook well, but I don't like going to houses. To avoid conflict, okay? Or having to say no. I know that there are people who know this, but I know that it is an effort for others if they want to please you … A friend, with whom I did not speak for two years, tells me: I have bought organic eggs; and I say: since when does a supermarket have organic eggs? Then I went to his house and it turns out that they were free-range eggs, category one, and not zero, which is the true organic one …***(M_42)**.

#### 3.1.3 My purchase: assessing food reliability

All knowledge and ideas resulting from health socialisation, the willingness to search for information, and meticulous research converge at the moment of purchase. At this time, knowledge, thorough analysis, and decision-making based on food quality come into play. Notably, food is assessed from multiple perspectives (ingredients, organic farming, and natural processing, among others). Thus, the highest quality food is that which is grown and processed by oneself. This is considered natural food, as it is produced in clean, safe contexts without substances that harm its nutritional benefits. Additionally, it is recommended to adhere to the guidelines set by the network of food commodification, ensuring the quality certification required by the community of eco-conscious consumers. Furthermore, food is purchased based on the precepts of naturalness and environmental awareness, seeking and, if necessary, visiting exclusively healthy and sustainable food establishments, stores, and supermarkets.

*Quality food … as it was in the past, it was not processed or anything … nothing mixed with chemicals or strange things; It was all natural. It was raised completely naturally, with animal manure … When you drank milk, it was real milk; when you ate fruit it was fruit. Now everything comes from greenhouses, and everything in cameras … They don't fool me with the issue of food. [ …] Something that has not been elaborated, processed … Cultivating you, right? For example, growing your own products, which you know are natural. It's not even what they sell me for being organic. […] It is clear that, if I am growing something of my own, I know that I am not going to put any of that in it***(E_42)**. *My boss currently does it. Sardines and other fish … Fish, even if they give you an organic can, the only thing that is organic is the oil. [ …] Eggs neither: it shows in the texture of the eggs. Do you know how the quality of an egg is measured? Well, when you break it, if the whole pan is filled with white … So when the white is very compact and the yolk is just on top, that's a super fresh egg***(M_42)**. *My father always said that phrase: these tomatoes are made of plastic, we don't buy these tomatoes, they are made of plastic. I said: plastic? Sure, and I imagined: oil. They have to put oil in it like they do in cars, you know? And I did not know, it did not enter my head. Of course, it's a greenhouse, it's hydroponic cultivation, which, I don't know if you've ever seen, a hydroponic cultivation greenhouse. […] They are plant hospitals***(ML_50/55)**. *Ecological, organic, biological is exactly the same. These are terms that, depending on the area, have been developed. Also in Latin America, be careful, organic … when they talk about organic it is not ecological, but they understand it that way because they have added manure fertilizer, but then they have cultivated with pesticides and everything, okay? I think that they are already differentiating, but according to the regulations and regulations, ecological, organic and biological are the same. The organic comes more from French, from the entire area of Belgium and such … […] In Spain the organic also began to be used, but in the end the organic ended up being standardized. [ …] One thing is the information I have about what an ecological is, and another thing is what I think an ecological should be, okay? An organic product should be a local, seasonal product, and free of all kinds of pesticides, chemical products; and done in an environmentally friendly way and in as long a period as possible, okay? No intensive production in greenhouses …***(ML_50/55)**.

#### 3.1.4 Moral burden

The meticulous, calculated, and demanding approach to shopping and examining food reinforces feelings of optimism and well-being for faithfully aligning with the condition. However, in specific moments of the women's experience, non-compliance with expectations triggers a sense of remorse or moral burden, leading to an anxious response and fleeting perceptions of the impossibility of continuing with the condition and fulfilling its demands.

This moral burden usually arises when failing to extend the condition within one's close social and affective environment (partner, children, and friends, among others) or to follow the routine (especially in the early stages of the condition). Over time, the moral burden, rather than being reactive to the immediate environment, becomes a political message for the entire world, promoting what is conventional and unhealthy and defiling the healthy condition.

*And you start to say … well, I don't care about this today … [ …] And you're out of control, because everything doesn't matter to you. There the mind does not control … because it also happens … [ …] Because it has happened to me … If I am not mentally well it is as if I had forgotten a bit about my diet. I didn't care***(E_42)**. *I don't know … it could be an external problem, or that you're going through a bad time in your life and it affects you mentally, and you're not well, you're not well, you can't concentrate. Still, it leads you to drop those healthy habits. You are more crushed by what is happening to you. [ …] If I am taking an excessive amount of sugars, carbohydrates, fats … I know that, in the long term, I am going to have health problems. And that is the weight of conscience that can fall on me if I do those excesses, because what worries me the most about all this is the disease***(E_42)**. *It requires a brutal transformation process. First you see it, you live it, and you think that it is the only thing and that the others are wrong; and you say that, and things happen. A friend stopped talking to me because I told her she was poisoning her children***(M_42)**. *Yes, it is true that sometimes it generates a bit of frustration, you can get depressed if you have all the information. I've only had one anxiety attack in my life and it was in the supermarket, okay? I had to leave once crying; I said: I'm going to have to go to a psychologist or something because it's not normal … […] You have to constantly regulate your emotions. You have to regulate your emotions when you live it this way***(E_42)**. *Eliminate fear, de-medicalize all these people who take many medications a day … I think even doctors would feel more satisfied, more psychologists … No, of course, but not like a psychiatrist taking pills and that's it. I don't know …***(ML_50/55)**.

#### 3.1.5 Compensation as a mechanism of channelling deviation

Moral burden, with its emotional and behavioural manifestations (anxiety, sadness, impotence, indifference, withdrawal, and evasion, among others), is alleviated or mitigated in gestures of compensation or regulation of the condition. This means a stronger commitment, with greater determination and firmer beliefs (as they have been, in some sense, questioned), to a healthy and sustainable diet as both a refuge and an instance of inquiry: how to be able to persuade oneself not to harm oneself with the information and lived reality, and how to convince the surroundings and the world at large.

*Because I know that later I'm going to do two hours of sports and that negative feeling goes away. [ …] I have had exceptions, but I know that on Monday I start doing sports again and I already compensate one thing with the other. Every time I go out to play sports, every day I feel very good; sport, and come home and take the corresponding diet that touches you after sports, and you feel great all day***(E_42)**. *But I didn't have all the information. The problem is that now it is no longer … It is no longer health, because I have already told you: I am not an example of health right now. That is to say, I have had to reduce cereals, I must reduce a series of things … Maybe I should pay a little more attention … the problem is that, when you are stressed, what you tend to do is cook less, and it is at reverse of what theory tells you, okay? But I say: yeah, pretty, but maybe you'll drink whiskey, and I don't drink alcohol, I've never had alcohol, and I don't like alcohol. So, I've never had alcohol, or wine, organic or non-organic, you know? I'm not going to have a drink either, I'm not going to have a drink either, you know? A series of escape routes that other people have. My escape route is to take bread with oil, and not cocaine***(M_42)**.

### 3.2 Becoming healthier

Following the model of *Health as expanding consciousness*, the female informants create their own health universe identifying the condition, synchronising body and mind (a body that increasingly craves health from natural and ecological sources and a mind that seeks, communicates, and recreates the surrounding world, in alignment with its prototype universe). This universe dilates and expands beyond the phenomenological field, in the shared experience of health and in the opposing or conventional experience. Accordingly, among the respondents, becoming healthier is the defining pattern of the health experience, which is understood by identifying patterns to discern ways of expanding consciousness.

*I think so … Women take better care of themselves than men. That in principle. Although, currently, also men … They are also getting into that plan to take care of themselves. Above all, if you are in a gym you realize that men take better care of themselves than before. Also because I think that women are also more presumptuous, more flirtatious, they like to feel good, look good and for others to see them well. We are more delicate when it comes to saying … Oops, I'm going to eat this, but not that. We have another idea … another idea different from that of the men, at least to me, specifically. [ …] But there are also others who do not care at all. They believe that in life you don't have to sacrifice so much in food, or suffer so much … that's what I hear, what they say, they tell me … That there are people who … Especially the men I've been with speaking, that they think it is suffering to live like this, because one wants to take care of oneself with food***(E_42)**. *The answer is … we want to be rich, we want it now and … well … Goodbye. That is to say, pure and simple egoism. So I don't want to. I do not believe in that. And a revolutionary act is to eat … and a revolutionary act is to decide that you eat organic and tell people about it. Invite people to try it; when people try it, they get hooked. […] So, how do you preach? By example … And I can't be a good example if someone … I don't even go in a McDonald's to piss; and it is political; that is, not to piss. Before, I peed on the door. But it's for ethics, you know? I mean, I don't like this model. […] So, with our consumption, we are deciding the type of life we want and the country we want, and the world we want. The moment it enters our heads that we have that power … It's that we have the power, no one else has it***(M_42)**. *All of this breaks the mold, because it is another way of life. If you want you can, the thing is that you don't want to. And in reality you don't know; You don't have the information, and it is very difficult to go against the current. [ …] What happens is that now there are so many of us against the current, that it is becoming standardized***(MA_40/45)**. *There are more and more things. There are more and more journalists interested in the topic, whether it's fashion, whether it's what you want, it doesn't matter. Even if they spell it wrong, it doesn't matter … They're dragging this out, suddenly you're in a bar and you hear some old men talking about ecology. […] So, there are many things that make people start to consider***(ML_50/55)**. *What they tell you out there: organically you cannot feed humanity. Well, then, very easy: there is a study, which I can also give you, that explains how it can be done, okay? A scientific investigation. […] With proximity ecology, that is, not with gigantic extensions, but with small extensions. […] We have to make a change … This beautiful word: a paradigm shift. If we want to continue consuming like crazy in this life, well … We are rejecting the other, we are losing the other; and we are aware. I say: be aware. People, when they become aware, even if it is little, are there. […] The seed grows alone, because it is common sense***(M_42)**.

#### 3.2.1 Highlighting benefits

Following a healthy and sustainable diet provides benefits at various levels: psychological and emotional, as a healthy lifestyle fosters positivity, optimism, and well-being with the body; sociological, as dietary and food preparation mechanisms and intricacies create a universe of complexity and intrigue driving curiosity and learning of the benefits and beliefs in those benefits. Accordingly, “highlighting” benefits also means projecting, assuming, and selling; that is, being immersed in the process of commodifying healthy food, strengthening it, and making it more robust, credible, and ethical, while arguing for its legitimacy and universal access.

*Physically, on the skin … I am a person who to this day has not had any major disease … [ …] It's obvious. Healthy eating … it is clear that you are going to have a healthier body, externally and internally. There is simply no more. I repeat: what you eat is what you are. There is simply no more. There is no more, I don't know … It shows in a person when they don't have a good diet, they reflect, they look physically … You avoid many illnesses. [ …] To cleanse the body, to release … To release all the remains that are in your body that are negative …***(E_42)**. *By being constant you manage to be well. In other words, it is not only accomplished mentally. I have experienced it. Of course, I feel good out of conviction, okay? But I have experienced it in my environment. The person I'm with is … He told me: I feel better, I get up better in the morning, I sleep better, my intestine doesn't sound***(M_42)**.

#### 3.2.2 Marketing as a pandemic resource of ideals

The female informants’ world of healthy eating evolves through expansion and contraction within their phenomenological field in their efforts to morally and politically influence their immediate environment and the world around them. They embrace this mission with mixed feelings of concern and compassion, dedicating themselves wholeheartedly and leading self-controlled lives filled with a strong belief in effecting social change in dietary habits.

Thus, the female informants regard the environment (even the world, through a generalisation/extrapolation of their immediate surroundings) that does not understand or support them as the real victim, highly vulnerable to the commodification of the conventional social image of health (which is not aligned with the ideal of natural and ecological health).

*It is clear that the world of food, through the media, is something that sells. It is something that sells, and they are taking advantage of the fact that there are more and more people who care about life, about leading a healthy life***(MA_40/45)**. *A way of wanting to sell something that is a lie, that is false … Deceive, deceive the consumer, that is clear. [ …] About products that tell you that you are going to lose so many kilograms in a month … That is a lie. I don't think so, I don't think so. You will never be able to believe anything that is sold to you in this sense, if you are not accompanied by something else: sports, advice … Which are not, I do not believe in what the media sells you on the subject of miraculous diets; that's what I don't believe. No, no, I don't believe in that. [ …] But it is that I do not let myself be guided by anything. I'm not looking for anything ideal, you know? I'm not copying myself; it's me. It's me, I don't let myself be influenced by anything they sell, by great bodies or anything like that. It's me. I know … It's in my head that it's like that***(E_42)**. *That's why the transgenics haven't won the battle, huh. Because people are scared. What happens is that they are selling it to us without labeling. [ …] All the animals that you consume are transgenic, because all the feed is transgenic. Soybean and corn, everything transgenic … Even the organic one is already contaminated. Pollen doesn't understand: let's see, you're transgenic, because I don't pollinate you. The pollen does not understand. So, all these things set trends***(M_42)**. *So, it is very difficult for a government to defend the ecological, because then you have to say that with the other you are poisoning humanity. […] Because the arguments in favor of organic are very simple: there are people who say that it is tastier, and I say that it depends. […] Organic crops, if they are greenhouse crops, are also harmed, because they are forced crops***(ML_50/55)**. *So, the power is in us … [ …] It is you who will begin to make waves in your environment. […] There is an ideological point that can help you; but you have to believe a lot in that ideological point, so that a transformation can be made. And that, even having that ideological point and that ideology, there are times when it is necessary to regulate …***(MA_40/45)**.

#### 3.2.3 Is the clinical eye of the family a stigmatising label?

The family sphere raises several questions in the female informants’ lived experience. The gradual and irregular expansion of the condition in the immediate surroundings and family entails focusing on their attention and prominence of their particular lifestyle, giving rise to both laudatory and derogatory and sarcastic opinions and considerations.

It is precisely within the family circle that the condition of a healthy and sustainable diet is engendered by the female informants and where, from one generation to another, the same dynamic is either transmitted or attempted to be established. However, that expansive, irregular, and intermittent gesture is precisely the mechanism or the pattern that sustains and maintains the seed of the condition. The family environment, smaller than the global or world environment, condenses and represents the roots of the condition: freedom and culinary mastery at home, in the security of trust, and in the warmth of companionship.

*Well, the family … They totally ignore me. It's like … I don't know, they think that I eat little, that I don't eat well or that I'm underweight. That's what I have from my family. And friends … It depends on what kind of friends … If they are the ones who move in the same circle with me, they think I do very well. If not, if it's not from that same group, they think I'm obsessed***(E_42)**. *Important in my life right now is also my environment, my family. And yes, they are related to food, because I want them to also lead the same healthy life; and I try a little to get them to take it. If it's not the same as mine, then similar. Because you can't force people either, right? If they don't want to, because they have other tastes or they like other things. But I try, for me it is important, that the family also leads a good nutritional life***(E_42)**. *It's a fight. It's a struggle, because, I repeat: I give a family member a plate of vegetables, and without fat, and without salt, and without oil and all those little things, right? And sometimes they look at you with a poker face and tell you: not me, I'm still hungry. They want a good stew. That's trying, which in the end is achieved little by little, and I say: try it … [ …] At home is where you can always control your diet well***(E_42)**. *But, going further, if you are an unbalanced person, your environment will be unbalanced to support you. And everything you will generate around you will be imbalance. […] But your day to day … your day to day is what decides how the world changes. And if you're going to eat there on Saturday you're not going to cause drama … Well, look, that's it, if the least I care about is the food, even if I enjoy it. But the least I care about when I go out is the food. It's being with friends, just like that, and that's it, and that's it. Know? I'm not going to overwhelm myself with this conventional game … Before, yes. Not now***(MA_40/45)**. *You feel guilty. It's that I see it that way. When they criticized me, it bothered me a lot. But then you analyze it and … Well, it just makes you scream in the face, but you think and … I believe in the mirror theory; you analyze it and you realize. But then it happens that they see how you live, they see what you do, they see that you're not that weird either …***(M_42)**.

## 4 Discussion

As for expanding the consciousness of healthy eating, a study has defined the orthorexic profile as an individual from a high socioeconomic level and with higher education who is well informed and investigates everything about food and a healthy and sustainable diet, in line with the themes “Researching and gathering information” and “My purchase: assessing food reliability” [20]. The themes “Moral burden” and “Compensation as a mechanism of channelling deviation” are related to the predispositions for regret and more extreme/condition-affirming behaviours if dietary expectations are not strictly met, as reflected in the original work of Steven Bratman [8].

The recent increase in literature, other documents, relevant clinical experience, and research studies underscores the significance of the topic at hand: the cult of the body and various trends in eating behaviour are evident phenomena in Western society [23]. A good physical appearance and health are synonymous with happiness, and by extension, people are drawn to healthy products and activities [21]. This contextual depiction of the orthorexia nervosa phenomenon encompasses, more or less simultaneously, several processes that converge towards a common outcome: the culture of health and, even more so, the culture of *healthism* (the concept of health as an individual responsibility, understood as an end in itself, as a virtue which should be valid for each and every human being, rather than a means to achieve life goals) [42].

The comfort and well-being that human beings predisposed to orthorexia nervosa express when planning and preparing a menu or a dish exclusively consisting of organic, sustainable food or products with health certificates are often associated with “vegan circles”, macrobiotics and nutritional patterns influenced by philosophies such as the blood type diet, the zone diet, the elimination diet, and the hyperactivity or attention deficit disorder diet [43].

In turn, the issue of environmentalism is unavoidable. Here, therefore, the ideology of environmentalism should be discussed according to the success of its three variants in challenging the anthropocentrism perpetuated by the Western worldview: environmentalism, conservationism, and true environmentalism [44,45]. Having witnessed the degradation of the natural ecosystem by the social ecosystem, environmentalists believe that the role of nature must be emphasised and that the importance of human beings and their social environment must be relativised. Indeed, since humans have inhabited the world, they incessantly embraced and censored different parts of it, distinguishing between a positive nature that is part of the system (agriculture, zoos, protected species, and natural parks) and a negative nature that becomes an ecosystem (battles against pests, harmful animals, viruses, and bacterias). At present, this labour of domestication and expulsion allows us to assert that nature has been almost completely socialised and that the emergence of environmentalism seems to coincide with its disappearance, thus explaining Beck's statement: “Nature is not nature, but rather a concept, norm, memory, utopia, counter-image. Today more than ever, now that it no longer exists, nature is being rediscovered, pampered” [46].

The contextualisation of environmentalism is likely an agent of nature conversion into a new narrative. Nature is presented as a religion by offering a set of interpretive moral values that influence ethical action. These values simultaneously serve as judges of human behaviour and even fill or eliminate the political void created by materialism and consumerism. From this perspective, environmentalism may synthesise two major narratives (the religious and the political), presenting itself as an approach that conveys a “saving message”: environmentalism as an ideology of capitalism and as a new religion, with a mission to mitigate, rather than vindicate, the aspirations and crises of capitalism [46,47].

Currently, eating healthy carries a clear political connotation. Healthy living has actually been an ideological representation of a specific social group: the upper-middle class. Therefore, being healthy is not equally accessible to everyone, which raises a strongly influential question: if only the affluent or wealthy can afford a healthy life, is healthy eating a personal and societal matter in which the healthcare sector has no say? Would equitable access to healthcare solve this issue of access to healthy eating so that everyone could have a healthy lifestyle? In Western culture, until the late 17th century, food was associated with the prevailing conception of health. However, with modernity, food was removed from popular medical knowledge. Not until the 20th century did food return to the corpus of medical knowledge. Now, food is regarded as a way of promoting health and preventing disease; now, food is medicine [48].

The theme of the concern about what is healthy (natural and ecological) remains, nevertheless, controversial: some written documents (opinion articles), from a sociological and anthropological perspective, place this predisposition within a marked spectrum of criticism. Examples such as *Contra la tontería de lo natural* [Against the foolishness of natural food] [49] and *El cuerpo, escenario de poder* [The body, a stage of power] [50] depict the discussion and lack of consensus regarding the representativeness (in terms of adequacy to experience) of orthorexia nervosa as a significant construct among more reasoned opinions and considerations on whether orthorexia nervosa is, actually, a health issue (biological, social or cultural) [[51], [52], [53]].

The limitation that arises when comparing studies on the same phenomenon (orthorexia nervosa) must be acknowledged because the studies published to date have been primarily quantitative and have not been approached from a constructivist paradigm. Nevertheless, there has been a gradual, and in recent years more pronounced, increase in epidemiological studies reporting the prevalence of orthorexia nervosa, assessed by applying Bratman's diagnostic test [22,[54], [55], [56], [57], [58], [59], [60]]. However, when analysing the individuals' subjective experiences, a clear instability is identified, generating ambiguity. For this reason, the inadequacy of this construct should be considered, both as a term and as a meaning whose attributes include “social stigmatisation” and “obsession as a defining characteristic of the condition”. Accordingly, the present study leaves room for questioning the view of healthy eating as a disease.

One of the latest publications of Steven Bratman, the author who coined the term and introduced the diagnostic proposal, opens the horizon of the defining characteristics of the condition beyond mere obsession, indicating that healthy eating operates within a process and fluctuates between phases of varying intensity [61].

Orthorexia nervosa, whether manifesting as a mere concern or an extreme obsession with healthy eating, borders on obsessive-compulsive disorder, anorexia, and selective eating disorder [[62], [63], [64]]. This study may help clarify those boundaries and gain a deeper understanding of living conditions worried about healthy eating. Some of the published studies determine the severity of the orthorexic condition as a risk of malnutrition or a range of metabolic dysfunctions for strictly adhering to a diet considered healthy by those experiencing such symptoms [65]. However, in light of the findings of this study, the severity of the condition should be questioned. Specifically, the benefits of maintaining the condition should be assessed in relation to its costs (such as hypovitaminosis, fixations, selectiveness, “manias,” and malnutrition, among other consequences) and whether, in the long term, the well-being generated by the condition outweighs its “harmful consequences."

Perhaps an aspect often overlooked, yet clearly manifested, is the presence of dysfunctional social relationships. Even the loss of sociability, generated by isolation and distance from the (dominant) world of conventional food, results in lost friendships and broken family ties, in line with the theme “Is the clinical eye of the family a stigmatising label?” [43].

Expanding the consciousness of healthy eating is a constant exercise of the condition throughout its phenomenological field (from its inception to its evolution), as a personal (seeking the harmony of absence of disease) and social (seeking congruence and global respect for nutrition, for the body and for society) struggle: “everyone must eat healthy” because it entails personal growth and constructs a fully conscious and feasible subjective universe of health.

Taking a holistic perspective of the healthy eating phenomenon enables a more in-depth approach to emerging and complex predispositions of human beings. Thus, taking care of human beings and their conditions, beyond whether they are healthy or pathological, begins with a modest approach, gradually leading to a comprehensive exercise in understanding.

Regarding the limitations of this research, the necessary trust and rapport took a long time, having to ignore data sources that were again not accessible and assuming laborious analytical work simultaneous to data collection. In this sense, the sampling was more intentional than theoretical and was limited to a single area.

This research had a necessary subjective component that, although it favored the theoretical and interpretive development of the study, sometimes questioned its consistency with the main objective of the study. The reason for this is that the study turned out to be more interpretive than descriptive. This study was limited when it came to extrapolating the findings to other contexts, because it has an unavoidable cultural component, which can vary according to gender, identity, religion and education.

## 5 Conclusions

The phenomenological field of healthy eating finds continuity and consistency in expanding consciousness: “Researching and gathering information” within “Health socialisation”, “My purchase: assessing food reliability”, “Moral burden”, and “Compensation as a mechanism of channelling deviation”.

“Becoming healthier” is the exercise that identifies and advances irregularly, expanding and contracting the condition of healthy eating in the environment through “Marketing as a pandemic resource of ideals”, “Highlighting benefits”, and “Is the clinical eye of the family a stigmatising label?”.

The concept of expanding consciousness in relation to healthy eating encompasses a continuous process where individuals not only seek information about what to consume but also actively participate in constructing a sense of identity through their food choices. By “Researching and gathering information”, people are not only educating themselves about what is considered healthy but are also contributing to a process of “Health socialisation”. In this context, knowledge about food becomes a form of social capital, where the ability to assess the reliability of food is linked not only to physical health but also to a moral construction of identity. The “Moral burden” associated with eating can lead to feelings of guilt or the need for compensation in cases of transgression, reflecting how eating takes on a deeper ethical and emotional dimension.

This cycle of *expansion and contraction* in the pursuit of optimal health does not occur in isolation. It is constantly influenced by external forces, such as marketing, which acts as an idealised resource that promotes perfection and well-being, particularly in global crisis contexts such as pandemics. Marketing campaigns often highlight the benefits of certain products or eating habits, but they also contribute to the creation of unattainable or stigmatising standards, especially when these narratives are integrated into family dynamics. The clinical eye of the family can become a mechanism of control or judgement, especially if certain behaviours or food choices deviate from what is perceived as “right”. This introduces an element of stigmatisation that affects both the ‘psychological and social health’ of individuals, creating an environment where food decisions are constantly scrutinised.

The definition of *orthorexia nervosa* as a disease also requires critical reflection. Although this term does not have formal recognition in traditional biomedical criteria, its use in social contexts can have significant implications. People who exhibit obsessive behaviours towards “pure” or extremely healthy eating often experience socialisation difficulties due to their idiosyncratic food ideologies. The way these individuals experience food differs from social norms, which can lead to their stigmatisation. Moreover, the use of the term orthorexia nervosa, resonating with other conditions such as anorexia or muscle dysmorphia, can be perceived as offensive by those who suffer from these conditions, as it suggests a pathologisation of behaviours that, from their perspective, are considered healthy or moral. This reflects a tension between biomedical definitions and cultural constructions of food health.

From a nursing perspective, understanding the phenomenon of healthy eating requires a holistic and global view that considers not only the physical aspects of nutrition but also the affective and psychosocial processes that influence the eating experience. Women, in particular, may attribute deep subjective meanings to their food choices, as these are often linked to social and family expectations about care and responsibility.

Qualitative evidence is fundamental in exploring these meanings, as it allows access to experiential discourses that reveal how individuals live and perceive their relationship with food.

This focus on subjective experience also allows for a deeper analysis of the cultural processes underpinning food practices. The culture of care surrounding food does not only refer to the provision of healthy food but also to how emotional and psychological health is cared for through eating. In this sense, a nursing approach that addresses both the affective and social dimensions of eating can offer a more integrated care. This means recognising that health is not only measured in biomedical terms but also in terms of emotional and social well-being.

Finally, for nurses, this holistic approach requires cultural and emotional sensitivity to the dynamics surrounding food. Understanding the subjective meanings of eating allows for a more empathetic and effective intervention that is not limited to promoting healthy habits but also supports individuals in their process of self-definition and in their relationship with others. The challenge lies in balancing the promotion of physical health with respect for the diversity of experiences and food practices, avoiding stigmatisation or the imposition of rigid standards of healthy eating.

## CRediT authorship contribution statement

**Ginés Mateo-Martínez:** Writing – review & editing, Writing – original draft, Visualization, Validation, Supervision, Resources, Project administration, Methodology, Investigation, Funding acquisition, Formal analysis, Data curation, Conceptualization. **Antonio Vázquez-Sellán:** Writing – review & editing, Writing – original draft, Methodology, Formal analysis, Data curation, Conceptualization. **María Luisa Díaz-Martínez:** Writing – review & editing, Writing – original draft, Visualization, Validation, Investigation, Formal analysis, Conceptualization. **María Carmen Sellán-Soto:** Writing – review & editing, Writing – original draft, Visualization, Validation, Supervision, Resources, Project administration, Methodology, Investigation, Formal analysis, Data curation, Conceptualization.

## Data availability statement

Data included in article/supplementary material is referenced in the article (Annex 1).

## Ethical aspects

This research follows the principles of the Declaration of Helsinki (2024) and the recommendations of the International Committee of Medical Journal Editors (ICMJE) for the conduct, presentation, editing and publication of academic works in medical journals.

## Ethical approval

Research Ethics Committee of the Faculty of Medicine of the Autonomous University of Madrid (CEI-69-124).

## Funding details

This paper is not supported by any funding or other type of economic support.

## Declaration of competing interest

The authors declare that they have no known conflict of interests or personal relationships that could have appeared to influence the work reported in this paper.

## Footnotes

1 Acronyms: (1) Initials of the name; (2) Age (years).
