# Supplementary material for: Orthorexia nervosa: Healthy habit or pathology? Experiences and expansion of the consciousness of the correct diet
Source: Heliyon. 2025 Jan 27;11(3):e42254. doi: 10.1016/j.heliyon.2025.e42254 (PMC11815929; doi:10.1016/j.heliyon.2025.e42254)
Supplement: Multimedia component 1 [file mmc1.docx]

**Data analysis**

**Noetic-noematic correlation (natural linguistic units, codes, discursive weight and order of appearance).**

| **NOEMAS**  **(natural linguistic units)** | **NOESIS**  **(codes and discursive weight)** | **NOESIS (order of appearance)** |
| --- | --- | --- |
| 'One thing has led to another' 'Since I was very little' 'My mother instilled it in me' 'A child's health problem' 'I got into healthy eating' | *Origin of the process 3, 3→ 6*  *Justification of the condition 2, 1, 2, 3→ 8* | *1*  *2* |
| 'Constant'  'Constancy'  'Advantages'  'Delay possible illnesses'  'Delay aging'  'You feel better' | *Characteristics of the condition 2, 2, 1, 1, 1 → 7*  *Purposes of the condition 3, 1, 1, 3, 3, 8, 2 → 21*  *Positive sensations and feelings 1, 1, 1, 5, 5 → 13* | *3*  *4*  *5* |
| 'Mentally'  'More positive' | *Involvement of the mind 1, 1, 2 → 4* | *6* |
| 'They eat anything'  'they don't care'  'Other people tell me that it shows'  'They see me and tell me that I eat well' | *The rest: those who are not 3, 1, 4 → 8*  *Those in my circle 2, 1, 3 → 6* | *7*  *8* |
| 'In the physical, in the skin'  'No major illness' | *What is considered a benefit 2, 2 → 4* | *9* |
| 'Sports center'  'Where else are those types of conversations found'  'The issue of food'  'Sport and food go together' | *Social place of the condition 3*  *Food status links 1, 1→ 2* | *10*  *11* |
| 'At home'  'You can control a diet well'  'Outings to restaurants'  'Some temptation' | *Place of full development of the condition 1*  *Control 1, 2 → 3*  *Disadvantages of leisure 2, 1→ 3* | *12*  *13*  *14* |
| 'Food world'  'Something that sells'  'They are taking advantage of the pull'  'The media' 'More people who are concerned about leading a healthy life' | *The world of food 1*  *Commercialization through the media 3, 1, 1→ 5*  *Expansion of the condition in number of people 1, 1→ 2* | *15*  *16*  *17* |
| 'Things I hear'  'I totally disagree'  'Wanting to sell something that is a lie, that is false'  'Deceive the consumer' | *Disagreement with the media 3, 3 → 6*  *Feeling of deception 1, 1, 1, 1, 1→ 5* | *18*  *19* |
| 'Eating healthy makes me feel very good'  'I know that I am a healthy person'  'Exceptions'  'I shouldn't have done it' | *Awareness of one's own state of health 1, 1→ 2*  *Repentance or burden of conscience 1, 1→ 2* | *20*  *21* |
| 'Two hours of sport'  'I'm losing that negative feeling'  'Compensation'  'Sport'  'The corresponding diet'  'You feel great all day' | *Sport as compensation 4* | *22* |
| 'They completely ignore me'  'They think that I eat little, that I don't eat well or that I'm underweight' | *Family perception 2* | *23* |
| 'Those who move with me'  'Same circle'  'It depends on what kind of friends' 'If not, they think I'm obsessed' | *Stigmatization 1* | *24* |
| 'You have to be very sure of what you are going to eat'  'quality'  'They are deceiving you'  'They tell you that it is the best quality they have'  'The best on the market' 'You start doing a little research'  'It doesn't carry anything good'  'They are poisoning you'  'Processed foods'  'They sell them to you as healthy and they are not'  'You must be very careful'  'Nowadays, there are few natural foods' | *Food quality 1, 1→ 2*  *Research and search for information 1*  *Unreliable food composition 1, 1→ 2*  *Unreliable characteristics of foods 1, 3, 2 → 6*  *Perception of consumption of conventional foods 1*  *Availability of natural foods 1* | *25*  *26*  *27*  *28*  *29*  *30* |
| 'Quality food'  'As of old'  'It wasn't processed at all'  'Nothing mixed with chemicals'  'With strange things'  'Everything was raised naturally'  'Animal fertilizers'  'When you drank milk, it was real milk'  'Everything about greenhouses'  'Everything in cameras' | *Characteristics of natural food 6, 4, 1→ 11*  *Genuine food 1, 1, 3 → 5* | *31*  *32* |
| 'Important in my life'  'My environment'  'My family'  'May they also lead the same healthy life'  'I'm the one who deals with food at home'  'The one who cooks'  'You can't force people either'  'I try, for me it is important, for the family to also lead a good nutritional life' | *Important vital aspects 3*  *Need to transmit to the close environment 3*  *Culinary mastery and responsibility 2* | *33*  *34*  *35* |
| 'It's a fight'  'Dish made from vegetables'  'Fat-free'  'Without salt'  'Without oil'  'They look at you with a face'  'Attempt'  'It is achieved little by little' | *Perception of the family's attempt to involve them 3* | *36* |
| 'Fresh'  'Reading the labels'  'What they contain'  'Too many carbohydrates'  'Too many sugars'  'Something that doesn't convince me'  'I don't consume it' | *Disposition in purchasing food 3*  *Less recommended foods 2*  *Reluctance to consume certain foods 1* | *37*  *38*  *39* |
| 'Not reliable' | *Food reliability 1* | *40* |
| 'I know I have to eat them too'  'They are necessary'  'You have to know the amount'  'Anything goes on a diet'  'Always in what quantity you take it' 'Do not cause excesses'  'A diet is variable'  'Need to eat a bun'  'Burden of conscience'  'I don't do it every day' | *Awareness of the need, despite not being recommended 2*  *Importance of the proportion of quantities 3, 1→ 4*  *Awareness that each food affects the entire organism 1*  *Diet variability 1, 1→ 2*  *Awareness of the consumption of less recommended foods 1* | *41*  *42*  *43*  *44*  *45* |
| 'Everything has a limit' | *The limit of the condition 1* | *46* |
| 'I'm doing well for my health'  'Do not consume these prohibited products'  'In the long term, I will not have a health problem'  'Burden of conscience'  'What worries me most about all this is the illness' | *Concern feedback 1* | *47* |
| 'You are what you eat' | *Identifying with food 1, 1→ 2* | *48* |
| 'Balanced diet'  'Five meals a day'  'Recommended amounts'  'The variety you need'  'Don't always eat the same thing'  'Balanced'  'Everyone knows it'  'Not everyone puts it into practice' | *Balance 2*  *Knowledge that the world has about healthy food 1*  *Absence of practice by the entire population 1* | *49*  *50*  *51* |
| 'Something that has not been elaborated, processed'  'grow yourself'  'grow your own products'  'you know thoroughly'  'they are natural'  'Not even what they sell me is organic, I believe it is natural' | *Self-management and self-production of food 2, 1→ 3*  *Awareness of the naturalness of the product 2*  *Distrust in the naturalness of food 1* | *52*  *53*  *54* |
| 'feel positive'  'it makes me happy'  'You feel happy'  'a mind-body'  'at ease with herself'  'that feeling is happiness'  'all related a little'  'body, mind' | *Inseparable binomial: mind-body 4* | *55* |
| 'mentally'  'control your schedules, your meals' 'mental work'  'self-control'  'uncontrolled'  'the mind is not controlling' | *Lack of provisional control 2, 3 → 5* | *56* |
| 'Healthy eating'  'a healthier body'  'externally and internally'  'what you eat is what you are'  'is reflected'  'you can see him physically'  'You avoid many diseases' | *Healthy eating 1* | *57* |
| 'The mind'  'Importance, all'  'I relate everything to the mind'  'It's all mentally' | *Consciousness and mind 4* | *58* |
| 'I don't let myself be guided by anything'  'I'm not copying myself'  'It's me'  'I don't let myself be influenced by anything they sell'  'great bodies'  'It's in my head' | *Reluctance to the media 5*  *Awareness that the condition is self-produced 2* | *59*  *60* |
| 'More women'  'Today, men have also put themselves'  'In that plan of taking care of yourself'  'Men take care of themselves more than before'  'Women are also more conceited'  'More flirtatious'  'He likes to feel good, look good'  'Others see it well'  'We are more delicate'  'We have another idea' | *Gender differences 7*  *Recent increase in condition in men 3* | *61*  *62* |
| 'Way to choose this type of life'  'For health reasons'  'Health'  'You feel great'  'Physically'  'You are healthy'  'Are you OK'  'You feel young'  'Not catch a disease'  'One of these diseases'  'Because of not having had healthy habits'  'Not taking care of yourself'  'If an illness has to come, it will come'  'The person who does not lead a healthy life will be more likely to suffer'   'Major illnesses due to not eating' | *Illness as a consequence of poor diet 5* | *63* |
| ‘…am I in the world of that kind of people?’  'Something may be that I identify with' | *Identification with the diagnostic proposal 2* | *64* |
| 'Obsession is not'  'It's worry' | *Confrontation of the diagnosis 2* | *65* |
